# The role of socio-demographic and health factors during COVID-19 in remote access to GP care in low-income neighbourhoods: a cross-sectional survey of GP patients

**DOI:** 10.1186/s12875-022-01887-5

**Published:** 2022-11-19

**Authors:** S. Sana, J. Kollmann, T. Magnée, I. Merkelbach, S. Denktaş, P. L. Kocken

**Affiliations:** 1 Erasmus School of Social and Behavioural Sciences, Erasmus University Rotterdam, Rotterdam, Netherlands; 2 Senior researcher, Nivel, Utrecht, Netherlands

**Keywords:** General practice, Primary health care, Access, Care use, COVID-19, Low-income population, Healthcare disparities

## Abstract

**Objectives:**

Remote consultations were common in general practice during the COVID-19 pandemic. This approach may have affected access to GP care for people with low socio-economic status: this group has a high prevalence of chronic conditions and a higher mortality rate due to COVID-19. This study explores the association of sociodemographic and health factors with the decision to contact a GP practice, and care utilisation, among patients in low-income neighbourhoods in the Netherlands.

**Design:**

Cross-sectional survey study.

**Setting:**

General practice in low-income neighbourhoods in the Netherlands.

**Participants:**

Patients from low-income neighbourhoods were selected from fourteen general practices on the basis of ethnic background, chronic disease or health literacy. Participants were stratified according to categories of these background characteristics to obtain equal numbers per category. A total of 213 surveys were retained for analysis.

**Main outcome measures:**

Need for GP contact, decision to contact a GP practice, and GP service utilisation.

**Results:**

Forty-five percent (*N =* 88) of the participants experienced health problems for which they wished to consult their GP at the start of the outbreak of COVID-19. A majority of them (81%) had contact with a GP service. The need to contact the GP was significantly associated with financial difficulties (OR 2.20 CI (1.10 to 4.39)). An interaction effect was found of health literacy with concerns about COVID-19 with in respondents with low health literacy a significant association between concerns about COVID-19 and a need for a GP appointment (OR 5.33 CI (2.09 to 13.59)) and absence of a significant association in the higher health literacy group (OR 1.14 CI (0.51 to 2.56)) *.* Moreover, 56% (*N =* 74) of the participants received remote care at least one time during the first wave of COVID-19. Female participants used remote care more often (OR 3.22 CI (1.57 to 6.59)) and participants aged 50 and over used remote care less often (OR 0.46 CI (0.21 to 0.97)).

**Conclusion:**

Many patients in low-income neighbourhoods were able to consult a GP, often remotely. However from the equity perspective, access to GP care should be safeguarded for patients with health problems, financial difficulties and low health literacy because of their greater need to consult a GP during times of crisis.

## Background

The COVID-19 pandemic has had a profoundly disruptive impact on society. Extensive measures restricting access to health care services were introduced in order to contain the spread of coronavirus. In particular, people with a low socio-economic status (SES), often in combination with a migration background, had a disproportionate health disadvantage during the COVID-19 pandemic, [1] resulting in higher mortality as a result of COVID-19 in this group [2].

In March 2020, GPs in the Netherlands and many other countries limited physical access to their practices in order to reduce the spread of coronavirus [3]. Most appointments were conducted remotely unless there was an urgent need for an appointment in person. At the same time, patients were also avoiding care settings because of the risk of infection with COVID-19 [4]. This led to an increase in consultations by telephone, email, video calls, and eHealth applications. We describe this type of consultation as ‘remote care’: all kinds of health care provision that substituted direct face-to-face contact at the practice [5, 6]. In the months that followed, GP practices gradually opened up, with more opportunities for face-to-face contact but with remote care still in place.

The use of remote care could have a disproportionate negative impact on people with low socio-economic status since GPs are the first point of contact and given the generally high utilisation of GP care due to a higher prevalence of major chronic conditions and lower self-efficacy in this group [7, 8]. The provision of remote GP care may affect access to care because remote care may not be an adequate way to deliver health services to disadvantaged groups. For example, there is a risk of digital exclusion in patients with a low socio-economic status and from migrant backgrounds due to a lack of digital literacy and access to technology, compounded by language barriers [4]. In general, low socio-economic status is a predisposing factor for contact with the GP practice and the utilisation of care, in addition to barriers to care and health care needs [9, 10].

In this study, we explore the impact of remote GP care on the decision to contact a GP practice and the utilisation of GP care among patients in low-income neighbourhoods in the Netherlands. We address the following research questions. Which proportion of respondents needed or wanted GP care at the outbreak of COVID-19 and which proportion obtained GP care? Which predisposing factors (such as gender, age and migration background) and enabling factors (such as access to care) and health need factors (like chronic disorders and concerns about COVID-19) were associated with the perceived need for GP care at the outbreak of COVID-19? What type of GP care utilisation (remote or face-to-face) was received during COVID-19 and which predisposing, enabling and health-need factors were associated with type of care utilisation?

## Methods

A cross-sectional survey was conducted of GP patients in low-income neighbourhoods in Rotterdam and the surrounding area during the first wave of COVID-19 (June to October 2020). The study was reviewed and approved by the Ethics Review Committee of the Department of Psychology, Education, and Child Studies of Erasmus University Rotterdam (file number 20–042, 18 April 2020).

All methods in this study were performed in accordance with the relevant guidelines and regulations.

### Procedure

We used convenience sampling to recruit patients. A group of fourteen general practices (*N =* 14) with nineteen active general practitioners (GPs) (*N =* 19) in our professional network were informed about the study and asked whether they would be willing to take part in the study. A low-income neighbourhood was defined as an area with a relatively high number of households at risk of poverty. Those households are defined by Statistics Netherlands as having an income of less than €1060 a month for a single person and €1460 for a couple [11]. GP care is covered by mandatory health care insurance for all Dutch citizens. There are no out-of-pocket costs for physical and remote GP consultations. The GPs gave explicit informed consent for participation in this study and for the recruitment of patients. The GPs were asked to include the patients from the following strata: (1) a migration background (Turkish, Moroccan or other) or non-migration background (Dutch) (2) low or medium health literacy as judged by the GP during regular interaction with the patient. (3) a chronic disease or no chronic disease. We aimed to obtain equal numbers of respondents for each group. The research team closely monitored the inclusion of patients until the pre-selected groups of patients were complete.

The GPs assessed the Dutch language proficiency of respondents. Respondents who were proficient in Dutch completed a written questionnaire handed out during consultation hours. The respondents who were not proficient in Dutch were called by an interviewer for a short interview (15–30 minutes) in the native language of the respondent. Respondents who completed the survey on paper at home and returned the survey by post gave consent on an informed consent form; for respondents who were called for a short interview their verbal consent was registered.

### Data collection

The questionnaire was based on theories about *access* to health care and *determinants of* health care use. We distinguished between the following stages in access to health care: the identification of health care needs, the decision to call on care services, the ability to access health care resources, and the actual utilisation of health care services [12]. Andersen and Newman’s behavioural model of health service use identifies the determinants of health care use on the basis of three factors, (1) predisposing individual factors such as gender, education and socio-economic backgrounds, (2) enabling factors such as practical barriers to GP care access and (3) health care needs, such as a medical condition (urgent or otherwise) [9, 10]. We studied these determinants from the perspective of the patient in the context of problem recognition, i.e. the patient’s identification of a personal problem as first step to be aware of a need for GP care, the decision to contact a GP practice and eventually GP service utilisation during the COVID-19 pandemic.

**Fig. 1:**
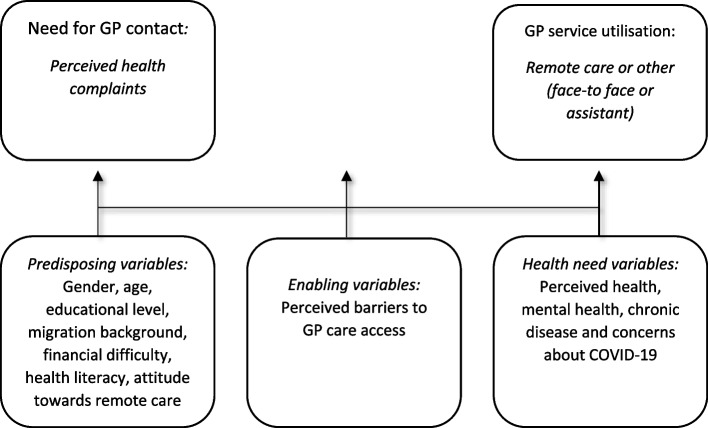
Conceptual model of GP service access

To identify the association of these determinants with GP care use, we summarised the different models in a conceptual model which explains the decision to contact a practice and the utilisation of GP care (see Fig. 1).

#### Survey questions

We used validated scales when available and assessed their reliability for the sample under study. The survey included scales used in prior research such as the Dutch nationwide Health Monitor Survey [13]. Questionnaire items were sometimes adapted to the population with low literacy for ease of reading. A team of experts consisting of four GPs working in low-income neighbourhoods was consulted to evaluate the questionnaire. Table 1 provides a summary of the questionnaire scales and items.

**Table 1:** Survey scales and items

Survey scales	Number of items	Examples of items and answer categories
Need for GP contact	2	‘Did you experience any health complaints in the first weeks of the COVID-19 outbreak for which you wanted to consult your GP?’ Answer categories ‘no’ or ‘yes’.
Decision to contact with GP practice	10	‘I tried to make an appointment, but it was cancelled by the GP’. Answer categories ‘no’ or ‘yes’
GP service utilisation	13	Which of the following ways of seeking care from the GP did you use during corona? ‘Consultation by phone’. Answer categories ‘no’ or ‘yes’.
Health literacy	2	‘How often do you have problems understanding your own health and complaints because you find the information about this, for example in brochures or on the Internet, difficult?’ Answer categories ‘always’, ‘often’, ‘sometimes’, ‘almost never’, ‘never’.
Attitude toward remote care option	2	What do you think about the fact that the GP offers the option of email consultation or a videocall instead of contact at the GP’s practice?’ Answer categories ‘very negative’, ‘negative’, ‘neutral’, ‘positive’, ‘very positive’.
Perceived barriers to GP care access	3	‘Which barriers to consulting your GP do you experience when you have health issues or problems?’ ‘There is a long wait when I try to make an appointment by phone’. Answer categories. Answer categories ‘no’ or ‘yes’.
Perceived health	2	‘How do you rate your health in general?’. Answer categories ‘very poor’, ‘poor’, ‘average’, ‘good’, ‘very good’.
Chronic disease	3	‘Do you have one or more chronic diseases (lasting longer than 6 months)?’. Answer categories ‘no’ or ‘yes’.
Mental health	2	‘The following questions are about how you have been feeling during the past four weeks.’ Answer categories ‘often’, ‘sometimes’, ‘rarely or never’.
Concern	2	‘How concerned are you about catching coronavirus?’. Answer categories ‘Not concerned at all’, ‘not very concerned’, ‘some concerns’, ‘very concerned’, ‘extremely concerned’.
Satisfaction	1	‘How satisfied are you with how your GP handled your health issues?’ Answer categories: ‘very dissatisfied’, ‘dissatisfied’, ‘neither dissatisfied nor satisfied’, ‘satisfied’ or ‘very satisfied’.

The outcome measures need for GP contact, decision to contact a GP practice and GP service utilisation were based on the model of access to care by Levesque et al. (Table 1) [12]. Need for GP contact was measured with one item on perceived health complaints for which the patient wanted to consult the GP in retrospect at the start of the COVID-19 outbreak. The decision to contact a GP practice by respondents who had a complaint was measure using 10 items on success or no success to receive a consultation from the GP for this health complaint. GP service utilisation was measured with 13 items about the ways in which they reached out to their GP during COVID-19, such as remote, face-to-face or contact with the GP assistant. The GP assistant is the entrance point to the medical practice and independently supports the GP with medical administrative procedures, such as administration of medicine subscriptions and keeping the agenda for consultation appointments. The assistant is trained to give medical advice to patients and to treat minor medical problems.

Questions about *health need factors* – perceived health, health complaints and chronic diseases – were derived from the national Health Monitor Survey [13]. Mental health was measured using the Mental Health Inventory (MHI-5) scale (Cronbach’s alpha = .85) [14]. Perceived concern about COVID-19 was measured using three items: (1) concern about the consequences of the COVID-19 crisis, (2) concern about contracting COVID-19 and (3) concern about infecting others with COVID-19 (α = .66) [15].

The enabling factor ‘perceived barriers to GP care access’ was measured using items based on the categories from the ‘barriers to treatment’ scale [16, 17]. Respondents could select multiple answers from a list of 15 items about whether they thought there were barriers and, if so, which barriers.

Moreover, respondents were asked about the predisposing factors gender, age, migration background, educational level and financial difficulties using the national Health Monitor Survey [13]. Health literacy was determined using two items adapted from Twickler et al. [18] and Wallace et al. [19] on problems with understanding information about your own health and completing a questionnaire about your health (r = −.61). We also included an item about patients’ attitudes toward remote care [20].

### Data analysis

We conducted factor analyses and reliability analyses of scales with three or more items, measuring mental health and concern. In case of two questionnaire items correlations were calculated and they were combined as single variables when correlations were high. Many answer categories of questionnaire items and scales were dichotomised around the 50th percentile. Moreover, the ten items regarding decision to contact a GP practice were recoded as a single variable with two categories: *consultation took place* or *consultation did not take place*. The thirteen GP service utilisation items were recoded as a single variable with two categories: *remote care* or *other* care, i.e., face-to-face care or contact with the GP assistant. Moreover, Firstly, descriptive statistics were calculated for the predisposing, enabling and need variables, and frequencies were given for females and males. Secondly, cross-tabulations were performed with need for a GP contact, decision to contact GP practice, and GP service utilisation as outcome variables, and predisposing, enabling and need variables as independent variables. Odds ratios were calculated for these bivariate associations. The analysis of a full logistic regression model with multiple independent variables was not possible for the outcome *need for a GP contact* because of multicollinearity of the independent variables. We did conduct logistic regression analyses with only two independent variables and the interaction term for those variables that were significantly associated with the outcome at the bivariate level. No logistic regressions were conducted for the outcome variables decision to contact *GP service* and *GP service utilisation* because of the absence of, or limited associations with, the independent variables. Any missing data was recoded into system missing and therefore excluded from the analyses. The survey data was analyzed using SPSS Statistics version 26.0.

## Results

### Respondents

A total of 213 respondents participated in the survey; 117 interviews were conducted by phone and 96 questionnaires were completed by the respondents on paper. We were able to include fairly equal numbers of respondents based on the presence of reported chronic diseases (*N =* 99), low health literacy (*N =* 102) and low educational level (*N =* 107). Respondents with a migration background were oversampled compared with respondents without a migration background (resp. 67 and 33%). Twenty-nine percent in total had a negative attitude to remote GP care. The data relating to enabling factors showed that 25% of the respondents encountered one or more barriers to access to a GP. In response to the questions about health needs, 60% expressed some or many concerns regarding COVID-19. Table 2 provides a complete overview of respondent characteristics, distinguished by gender.

**Table 2:** Characteristics of respondents for the total group and by gender

Variables	Total group (*N =* 213^a^)		Male (*n =* 92)		Female (*n =* 121)	
	N^2^	(%)	N^b^	(%)	N^b^	(%)
Gender						
Male	92	(43)	–	–	–	–
Female	121	(57)	–	–	–	–
Age						
< =49	82	(39)	31	(34)	51	(42)
50+	129	(61)	60	(66)	69	(58)
Education level						
Medium-high	104	(49)	44	(49)	60	(50)
Low	107	(51)	46	(51)	61	(50)
Migration background						
No	71	(34)	29	(31)	42	(35)
Yes	141	(66)	63	(69)	78	(65)
Financial difficulties						
No	162	(77)	63	(70)	99	(82)*
Yes	49	(23)	27	(30)	22	(18)*
Health literacy						
High	110	(52)	41	(45)	69	(57)
Low	102	(48)	51	(55)	51	(43)
Attitude remote care						
Positive	149	(71)	66	(72)	83	(70)
Negative	62	(29)	26	(28)	36	(30)
Perceived barriers						
No	159	(75)	73	(79)	86	(72)
Yes	53	(25)	19	(21)	34	(28)
Perceived health						
Very good or good	124	(59)	61	(66)	63	(53)
Very poor to average	88	(41)	31	(34)	57	(47)
Mental health						
Average-good	149	(71)	67	(74)	82	(69)
Poor	60	(29)	23	(26)	37	(31)
Chronic disease						
No	111	(53)	54	(59)	57	(48)
Yes	99	(47)	37	(41)	62	(52)
Concerns about COVID-19						
No	85	(40)	42	(46)	43	(35)
Yes	128	(60)	50	(54)	78	(65)

* p < 0.05

^*a*^ *total number of participants*

^*b*^ *n varies because of missing data*

#### Need for GP contact and decision to contact a GP practice at COVID-19 outbreak

**Fig. 2:**
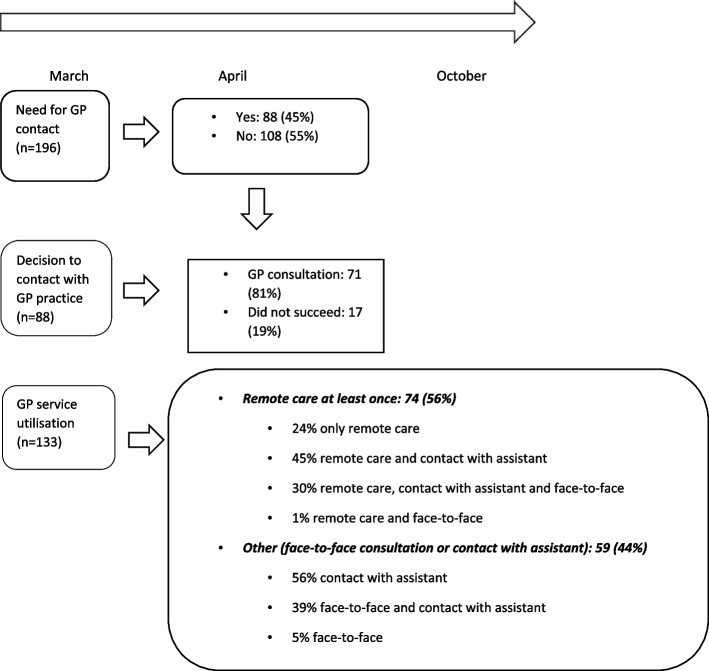
Proportion of respondents needing and utilizing GP care

Health complaints were experienced by 45% of the respondents for which they would have wanted to contact the GP during the early weeks after the outbreak of COVID-19 in the Netherlands (March–April 2020). Of this group, 81% successfully had contact with the GP. (Fig. 1). Moreover, over a longer period – the first wave of COVID-19 in the Netherlands (March–October 2020) – 56% of patients who had contacted a GP service at the time of completing the survey had received remote care at least once. Of the patients in this group, 24% had received remote care only. The rest of the respondents in this group had received remote care, either in combination with contact with the assistant (45%) or in combination with contact with the assistant and a face-to-face appointment (31%) (Fig. 2).

The predisposing factors *financial difficulties* and *health literacy* were associated with the respondents’ need for a GP appointment, which was higher in patients with major financial difficulties and low health literacy (OR 2.20 CI (1.10 to 4.39)) and (OR 1.88 CI (1.06 to 3.32)) respectively (Table 3). All measured health problems – perceived health, mental health, chronic disease and concerns about COVID-19 – were associated with the need to consult a GP. The strongest association was found with perceived health rated as very poor to average (OR 3.13 CI (1.73 to 5.64)). Moreover, using interaction analysis, we found a moderating effect of health literacy on the association between concerns about COVID-19 and need for a GP appointment. This association was significant for respondents with low health literacy (OR 5.33 CI (2.09 to 13.59)), but not significant for the respondents with higher health literacy (OR 1.14 CI (0.51 to 2.56)) (not shown in a table).

**Table 3:** Patients’ need for GP appointment GP and success in GP service utilisation at the outset of the COVID-19 outbreak, and remote care at least once (GP service utilisation) during the first wave of COVID-19. Bivariate associations with predisposing, enabling and need variables

Total group (*N =* 213^a^)	Need for GP contact (*n =* 196^b^)		Consultation (*n =* 88)^b^		Remote care (*n =* 133)^b^	
	**Yes**		**Yes**		**Yes**	
	N^b^ (%)	OR (95% CI)^c^	N^b^ (%)	OR (95% CI)^c^	N^b^ (%)	OR (95% CI)^c^
Gender						
Male	36 (43)	1.00	30 (86)	1.00	22 (39)	1.00
Female	52 (46)	1.16 (0.65–2.04)	41 (77)	0.57 (0.18–1.79)	52 (68)	3.22 (1.57–6.59)**
Age						
< =49	37 (47)	1.00	27 (73)	1.00	32 (68)	1.00
50+	51 (44)	0.86 (0.48–1.52)	44 (86)	2.33 (0.79–6.84)	42 (49)	0.46 (0.22–0.97)*
Educational level						
Medium-high	42 (44)	1.00	33 (79)	1.00	34 (60)	1.00
Low	45 (46)	1.09 (0.62–1.92)	37 (82)	1.26 (0.44–3.65)	38 (51)	0.71 (0.36–1.44)
Migration background						
No	27 (42)	1.00	23 (82)	1.00	20 (49)	1.00
Yes	60 (46)	1.16 (0.63–2.12)	47 (80)	0.85 (0.27–2.71)	53 (58)	1.46 (0.70–3.07)
Financial difficulties						
No	62 (41)	1.00	50 (81)	1.00	51 (53)	1.00
Yes	26 (61)	2.20 (1.10–4.39)*	21 (81)	1.01 (0.32–3.22)	22 (63)	1.53 (0.69–3.37)
Health literacy						
High	38 (37)	1.00	31 (80)	1.00	36 (55)	1.00
Low	49 (53)	1.88 (1.06–3.32)*	39 (81)	1.12 (0.39–3.24)	37 (55)	0.99 (0.50–1.97)
Attitude towards remote care option						
Positive	60 (44)	1.00	50 (82)	1.00	54 (57)	1.00
Negative	28 (49)	1.26 (0.68–2.33)	21 (78)	0.77 (0.25–2.35)	20 (53)	0.82 (0.39–1.75)
Perceived barriers						
No	64 (43)	1.00	51 (80)	1.00	54 (52)	1.00
Yes	24 (52)	1.47 (0.76–2.84)	20 (83)	1.28 (0.37–4.38)	20 (67)	1.82 (0.77–4.25)
Perceived health						
Very good or good	38 (33)	1.00	30 (79)	1.00	34 (51)	1.00
Very poor to average	50 (61)	3.13 (1.73–5.64)**	41 (82)	1.22 (0.42–3.52)	40 (61)	1.49 (0.75–2.97)
Mental health						
Average-good	55 (39)	1.00	47 (86)	1.00	52 (58)	1.00
Poor	32 (58)	2.15 (1.14–4.05)*	23 (72)	0.44 (0.15–1.28)	22 (52)	0.78 (0.37–1.64)
Chronic disease						
No	33 (34)	1.00	28 (85)	1.00	27 (50)	1.00
Yes	53 (56)	2.49 (1.39–4.45)**	41 (77)	0.61 (0.19–1.92)	44 (58)	1.38 (0.68–2.77)
Concerns about COVID-19						
No	26 (33)	1.00	22 (88)	1.00	27 (52)	1.00
Yes	62 (53)	2.30 (1.27–4.16)**	49 (78)	0.48 (0.12–1.83)	47 (58)	1.28 (0.64–2.58)

** p < 0.01; * p < 0.05

^*a*^ *total number of participants*

^*b*^ *n varies because of missing data*

^*c*^ *Unadjusted bivariate*

No significant association was found between the predisposing, enabling or need factors and the outcome of whether respondents who decided to consult a GP actually succeeded in having contact with a GP (Table 3). Patients who had an appointment with the GP and succeeded in arranging a contact early during the COVID-19 outbreak were mostly satisfied (96%) with the way the GP treated the patient. The few patients whose appointment was cancelled or who did not have a GP consultation despite their health complaint or worries were often very dissatisfied. The reasons they gave were not feeling heard or taken seriously and not being allowed to come to the practice.

#### GP care utilisation during the first wave of COVID-19

The predisposing variables gender and age were significantly associated with the type of GP service utilisation, with females using remote care more often (OR 3.22 CI (1.57 to 6.59)) and people aged 50 and over using remote care less often (OR 0.46 CI (0.21 to 0.97)) (table 3). In addition, neither the enabling nor the need variables were significantly related to the type of care utilisation. However, when we examined (very) poor perceived health compared with good perceived health (including average perceived health), we found people with poor to very poor perceived health had more frequent remote care (OR 6.65 CI (1.45 to 30.57)) (not shown in a table).

## Discussion

In this study, we explored the contribution of remote GP care in the decision to seek contact with a GP and in care utilisation among patients in low-income neighbourhoods during COVID-19. The study showed that most patients expressing a need for GP care managed to consult their GP despite the restrictive measures in place to contain the spread of COVID-19. A minority of patients were dissatisfied with GP care because of the limited access to their GP in the early stages of the pandemic.

Our finding that there was more need for GP contact at the start of the pandemic in patients with the predisposing characteristics financial difficulties and low health literacy can be explained by their poorer health outcomes and more frequent use of health care services in general [21–23]. We found that demand for care was largest in patients with low health literacy combined with a high level of concern about COVID-19. This study adds that patients with low health literacy particularly need contact with a health care professional to clear up their concerns during the COVID-19 health crisis.

We also found that patients susceptible to mental conditions and chronic health problems frequently felt the need to have contact with the GP practice during COVID-19. This concurs with research showing an increase in the number of mental health problems during the COVID-19 pandemic [24]. The association we found between poor or very poor perceived health and the use of remote care may be attributable to more intense concerns about health and therefore a stronger need to consult a GP remotely as a result of the pandemic. Patients with chronic health problems also had a need to for contact with the GP and may have been concerned with their health, probably because of the higher risk of morbidity and mortality associated with COVID-19 [25]. As in normal circumstances before the emergence of COVID-19, patients with mental and chronic health problems in low SES neighbourhoods wanted GP care but the circumstances during the health crisis may have exacerbated that demand.

We found no link between *barriers to care* access and the need for contact a GP or GP service utilisation. We did find that about 81% of the respondents who felt the need to contact a GP during the COVID-19 lockdown managed to consult a GP. This finding resembles that of a recent survey of telehealth in New Zealand [3]. Although it would appear that GP practices were, in general, accessible for patients from low socio-economic groups with concerns and problems with their health during the first period of lockdown, one fifth decided not to consult their GP or were not able to access the practice.

A substantial number of our respondents reported having received remote care at least once. A systematic review of telephone appointments with GPs showed that telephone advice alone was adequate for half of the calls [26]. We found that received remote care was significantly correlated with being female and being 49 years of age or younger. Female patients generally have more contacts with a GP than male patients [27, 28]. This may be attributable to the allocation of responsibility for care in average households to females [29]. Moreover, in line with other studies, we found that remote care is used more by younger people. Remote care also seems to be more accepted by younger people in general and to have been more accepted during COVID-19 [30, 31].

We found no association between educational level or migration background and the need for a GP appointment, decision to contact the GP practice or the type of GP service utilization. An additional analysis of correlates with the attitude of the respondents towards remote care did not show an association with migration background. However, patients with low educational level were more negative about remote care than patients with a higher educational level. This study showed that socio-economic – in other words, financial – difficulties, low health literacy combined with a high level of concern about COVID-19, and health factors would seem to be determinants of the need to consult a GP. It is important to secure the access to a GP for this group because they are more susceptible to mental and physical health problems. The urgency of implementing remote GP care during COVID-19 may have resulted in equal care provision and the equal utilisation of this type of care, regardless of the educational level or migrant status of patients. Nevertheless, the negative attitude towards remote care of patients with a low level of education should be taken account when providing remote care.

### Strengths and limitations

A strength of this study is the recruitment of respondents by GPs in the practices with patients from low socio-economic neighbourhoods. This probably increased the willingness to participate among people who are hard to reach, resulting in a significant high number of respondents with lower socio-economic status or a migrant background, groups which are usually underrepresented in studies. As GPs are familiar with their patients’ medical and personal histories, we were able to include a satisfactory number of patients with a migrant background, patients with chronic diseases or patients with a low educational level or low health literacy. GPs also recruited patients in less vulnerable circumstances who were also residents of low socio-economic neighbourhoods, and this allowed us to compare response groups.

Another strength of the study was that we conducted interviews in the native language of the respondents, allowing us to include patients with an inadequate command of Dutch. This approach made it possible to reach an underrepresented population and also prevented any misinterpretations or the loss of culturally sensitive information.

A limitation of our study could be the selection of patients with the convenience sampling procedure. This may have led to the selection of patients who were easy to reach, for example patients who do not avoid contacts with healthcare professionals, and probably an underestimation of results. On the other hand, it can be expected that vulnerable patients tend to have frequent contact with their GP, resulting in the participation of the targeted population [22, 23]. We asked respondents to report about decision to contact GP practice and service utilisation in retrospect and this may have led to recall bias. This loss of information may have been limited because we administered the questionnaire soon after the introduction of COVID-19 measures and restrictions. Another limitation may be the adaption of questions and scales to the target population. However, this adaptation for the limitations associated with the respondents’ low (health) literacy and the limited size of the questionnaire was needed to enhance the participation of the had-to-reach groups targeted by this study.

### Implications for practice

Vulnerable patients were in need of contact with the GP during COVID-19, and they were at a higher risk of morbidity and mortality due to COVID-19. Barriers in access to GP care can lead to an accumulation of somatic and psychological disorders in this group of patients. Actively targeting this group by providing sufficient access to health care and minimising barriers are important considerations during a health crisis.

This study found that patients with low health literacy in particular need contact with a health care professional to address their concerns. Literature shows people vary in their use of health technology, depending on the complexity of devices and level of health literacy [32–34]. Although we found use of remote care was not related to the patients’ extent of health literacy during the first wave of COVID-19, potential exclusion from use of substitute arrangements for GP contact in low digital health literate groups must be avoided beyond the period of COVID-19 pandemic. Therefore, it is important to monitor these patients actively during a health crisis. Investing in the improvement of people’s health literacy, for example by working with community health workers, will help patients to manage their concerns and enhance their self-efficacy to have contact with health care professionals.

Younger patients and females received remote care more often [27, 28, 35]. Besides consolidating effective remote care for younger patients, a targeted approach is recommended to the provision of remote care for certain other categories of patients, such as older patients. Nevertheless, GPs who are reluctant to provide remote care argue that it is not suitable for patients from low-income neighbourhoods and those with poor language skills [4]. We did not find an association between educational level or migration background and type of care utilisation. GPs may be biased in their views about the possibilities of using remote care for specific groups. It is important to explore and to take into account the concerns of GPs in this respect when targeting specific groups.

### Implications for research

In general, more research is needed into patients at risk of COVID-19-related problems and the suitability of remote GP care in low-income areas. Qualitative research is needed to explain the patients’ help-seeking behaviour and the views about remote care of care providers in general practice. Furthermore, the analysis of GP registration data is important to study the impact of COVID-19 on the utilisation of GP care during the different phases of the pandemic.

## Conclusion

During the first wave of the pandemic, physical access to a GP practice was minimal. For many patients from low-income neighbourhoods, contact with a GP was possible, often remote. However, access to GP care should be safeguarded for patients with health problems, financial difficulties and low health literacy because of their greater need to consult a GP during times of crisis.

## Data Availability

We are not able to make data underlying the findings described in our manuscript available without restriction, because of ongoing PhD project of one of the authors. Researchers interested in our dataset can contact the project leader Dr. PL Kocken (one of the authors) with reasonable request via email kocken@essb.eur.nl.
